# Analysis of Virus-Specific B Cell Epitopes Reveals Extensive Antigen Degradation Prior to Recognition

**DOI:** 10.3390/cells13131076

**Published:** 2024-06-21

**Authors:** Alvaro Ras-Carmona, Pedro A. Reche

**Affiliations:** Laboratory of Immunomedicine, Department of Immunology & O2, Faculty of Medicine, University Complutense of Madrid, Pza Ramon y Cajal S/N, 28040 Madrid, Spain; aras@ucm.es

**Keywords:** B cell epitope, linear B cell epitope, antibody, virus, structural analysis

## Abstract

B cell epitopes must be visible for recognition by cognate B cells and/or antibodies. Here, we studied that premise for known linear B cell epitopes that were collected from the Immune Epitope Database as being recognized by humans during microbial infections. We found that the majority of such known B cell epitopes are virus-specific linear B cell epitopes (87.96%), and most are located in antigens that remain enclosed in host cells and/or virus particles, preventing antibody recognition (18,832 out of 29,225 epitopes). Moreover, we estimated that only a minority (32.72%) of the virus-specific linear B cell epitopes that are found in exposed viral regions (e.g., the ectodomains of envelope proteins) are solvent accessible on intact antigens. Hence, we conclude that ample degradation/processing of viral particles and/or infected cells must occur prior to B cell recognition, thus shaping the B cell epitope repertoire.

## 1. Introduction

B cells recognize freely accessible antigens through their surface receptor (BCR), encompassing a membrane-bound antibody. Upon antigen recognition, and with the participation of secondary stimuli, B cells differentiate into plasma cells secreting antibodies that mediate humoral immunity [1]. B cells and antibodies do not recognize the antigen as a whole, but rather solvent-accessible portions known as B cell epitopes [2]. In protein antigens, these epitopes can be classified as conformational or linear. Conformational B cell epitopes, also known as discontinuous B cell epitopes, encompass residues that are not sequential but are in close proximity within the antigen tertiary (3D) structure [2,3]. Linear B cell epitopes, also known as continuous B cell epitopes, consist of sequential residues and, unlike conformational B cell epitopes, can be recognized by antibodies outside of the remaining protein [2,3]. Given the protein fold, it is often understood that most B cell epitopes are conformational [4].

B cell epitopes in protein antigens can be identified through different experimental methodologies. Arguably, the most accurate approach to identifying B cell epitopes is to solve the 3D structure of the relevant antigen–antibody complex, often via X-ray crystallography [5]. This approach serves to define *bona fide* B cell epitopes in native antigens, but its reach is limited by the need to have crystals of highly purified protein–antibody complexes. Therefore, researchers often turn to alternative approaches for B cell epitope mapping, such as determining antibody binding to overlapping synthetic peptides covering the protein antigen [5,6,7]. Unfortunately, these approaches are poorly suited to identifying conformational B cell epitopes, recognizing mostly linear B cell epitopes that may or may not be solvent accessible on the intact native antigen. Experimentally determined B cell epitopes are collected in dedicated databases [8]. Currently, the major B cell epitope repository is the Immune Epitope Database (IEDB) [9,10], which includes over 220,000 B cell epitopes (released 17 July 2023). Unlike other databases, the IEDB allows us to identify the context, including immunogens, relating B cell epitopes to cognate antibodies. In this work, we examined B cell epitopes targeted during the course of a natural infection in humans.

We found that the vast majority of B cell epitopes recorded at the IEDB as being recognized during the course of infection in humans are not conformational, but rather are linear and derived from viruses. Focusing on these virus-specific linear B cell epitopes, we found that a large portion (64.44%) of them reside in viral antigens that remain enclosed in host cells and/or viral particles, hidden to antibody recognition. Moreover, we also found that those mapped in visible antigens (e.g., the ectodomain of envelope proteins) have solvent accessibilities incompatible with recognition by antibodies occurring on intact cells or viral particles. Collectively, these results support the notion that extensive antigen degradation/processing must occur prior to recognition by antibodies, which shapes the B cell epitope and antibody repertoires. We argue that innate immune processes, such as phagocytosis followed by exocytosis, are likely involved in exposing hidden antigens and antigen fragments for antibody recognition.

## 2. Materials and Methods

### 2.1. B Cell Epitope Data Collection and Processing

B cell epitopes were obtained from the Immune Epitope Database (IEDB) [9,10] after a search for B cell epitopes with positive assays linked to infectious diseases in humans. In the IEDB, all data come from empirically derived assays, reflecting the interaction of an adaptive immune receptor with an epitope, which can be traced to relevant publications. Linear B cell epitopes cannot exceed 50 amino acids in length and must be tested as either immunogens or antigens. Linear B cell epitope assays include ELISA, western blots, radioimmune assays, and high-throughput multiplexed assays, among others. On the other hand, conformational B cell epitopes are generally determined by structural methods such as X-ray crystallography. All these assays have experimental error margins, but structural methods, which can pinpoint specific interacting residues, are considered the most precise of all. In the IEDB, the same B cell epitope can be linked to different assays. B cell epitope assay data were downloaded and parsed, selecting the epitope amino acid sequence (only unique sequences were considered), type of epitope (linear or conformational), and NCBI and UniProt accessions of source antigens. B cell epitopes that did not include NCBI or UNIPROT accessions, or did not have between 8 and 25 residues, were discarded. Amino acid sequences of antigens were obtained from UniProt using the relevant accessions. Linear B cell epitopes that did not match the sequence of source antigens were also discarded. Matching was verified through BLAST searches, as described in Ras-Carmona et al. [11]. CD-HIT [12] was used to cluster overlapping linear B cell epitopes (100% identity threshold). B cell epitope clusters were processed by selecting overlapping regions including 8–25 residues as representative linear B cell epitopes. An additional dataset consisting of conformational B cell epitopes was obtained as described elsewhere [13] from the known 3D structures of antigen–antibody complexes available in the abYbank/AbDb database [14]. Conformational B cell epitopes consisted of antigen residues with atoms contacting the antibody (≤4 Å distance).

### 2.2. Antigen Annotation

The taxonomy, sub-cellular location, and transmembrane topology of antigens were collected from protein records in the NCBI and UniProt databases, and recorded for every antigen. Taxonomy information was obtained from the NCBI taxonomy database [15] using the corresponding NCBI tax identifiers. The sub-cellular location of proteins and transmembrane topology of plasma membrane proteins were obtained from UniProt. Transmembrane topology was predicted using TMHMM [16] for protein antigens with plasma membrane sub-cellular locations but without information relevant to this subject.

### 2.3. Determining the Solvent Accessibility of B Cell Epitopes

Solvent accessibility was determined for linear B cell epitopes mapping to antigens with a known 3D structure and for conformational B cell epitopes defined after the 3D structure of antigen–antibody complexes. For linear B cell epitopes, the 3D structure coordinates of protein antigens were obtained from the Protein Database Bank (PDB) at Brookhaven [17], according to the relevant PDB codes in NCBI protein records. PyMOL Molecular Graphics System (Version 1.8 Schrödinger) was used to remove redundant molecules/chains co-occurring in crystallization units and to discard linear B cell epitopes that did not map to the relevant 3D structures. For conformational B cell epitopes, antibody chains were removed from antigen–antibody 3D structures prior to further analysis, which was also performed using PyMOL. The relative solvent accessibility (RSA) of protein antigen residues was obtained in % values according to the relevant 3D structure coordinates using NACCESS (version 2.1.1) [18], and B cell epitope RSA (eRSA) values (%) were subsequently computed using the following equation:

(1) $$\;eRSA=\frac{\sum_{i=1}^{i=N}{RSA}_{i}}{N}$$

where N is the number of residues in the B cell epitope, and RSA_i_ is the RSA of residues (i = 1, i = 2, …, i = N) included in the B cell epitope.

### 2.4. Graphics and Statistics

All plots were generated in RStudio with the help of specific R packages. Statistical differences between groups of B cell epitopes with regard to eRSA values were analyzed in RStudio using non-parametric one-sided Mann–Whitney U tests. *p* < 0.05 was considered significant.

## 3. Results

### 3.1. Characterization of B Cell Epitopes Targeted during the Course of a Natural Infection

**Figure 1:**
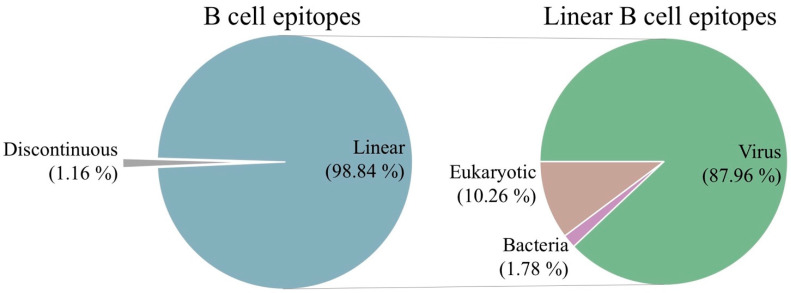
B cell epitopes targeted in humans during microbial infections. A total of 55,642 unique B cell epitopes with 8 to 25 residues were identified in the IEDB as being targeted during microbial infections in humans. Of those, 98.84% were linear B cell epitopes (**left pie chart**), with 87.96% mapping to virus antigens (**right pie chart**).

B cell epitope identification is of great practical relevance and is a matter of intense research activity. As a result, thousands of B cell epitopes have already been reported in the literature and recorded in databases, such as the IEDB. These B cell epitopes were identified under different experimental contexts, and here we sought to characterize those targeted in humans during infection. We identified and selected 55,642 such B cell epitopes from the IEDB, all including between 8 and 25 residues. This size range was chosen to limit the chance of considering B cell epitopes with excess residues that are not part of the B cell epitope. Interestingly, the vast majority of these epitopes (54,996, 98.84%) were reported as linear B cell epitopes (Figure 1). These linear B cell epitopes covered 4099 distinct antigen proteins, and we subsequently classified them according to the taxa of the source organisms (Supplementary File S1). As shown in Figure 1, 87.96% of the linear B cell epitopes (48,377 out of 54,996) were from viruses, and a mere 1.78% (979 B cell epitopes) and 10.26% (5640 B cell epitopes) were from bacteria and eukaryotic organisms, respectively. Given the abundance of virus-specific B cell epitopes, we focused on them in further structural and location analyses.

### 3.2. Analysis of the Visibility of Viral Antigens Encompassing Linear B Cell Epitopes

BCRs can only target antigens that are visible and solvent accessible. Therefore, we analyzed the location in host cells and viral particles of the 3010 antigens that encompassed the selected 48,377 virus-specific linear B cell epitopes, using the relevant UNIPROT annotations (details in Methods). It is important to remember that all viral proteins are produced by the host cells and hence have sub-cellular location annotations in UniProt. Taking these annotations into consideration, we classified viral proteins into four groups according to their visibility to antibodies on intact viral particles or cells. Non-structural viral proteins remaining within cells (in the cytoplasm, nucleus, endoplasmic reticulum, etc.) and structural proteins enclosed in viral particles were considered hidden from antibody recognition and included in a group that we termed Cell/Virus Enclosed. In addition, we considered three other major groups of viral antigens: secreted/extracellular proteins, consisting of non-structural viral proteins that are secreted by cells (e.g., proteins interfering with the immune response); envelope proteins, consisting of proteins that are located in the cell plasma membrane and subsequently move into the viral envelope; and capsid proteins, which form part of viral capsids. Envelope and secreted proteins can be considered readily visible to antibodies, while capsid proteins may or may not be visible for recognition depending on the type of virus. It is worth noting that proteins usually have more than one sub-cellular location annotated in UNIPROT, but none of the viral antigens classified into the Cell/Virus Enclosed group had a sub-cellular location that included the extracellular space, plasmatic membrane, or viral envelope.

**Figure 2:**
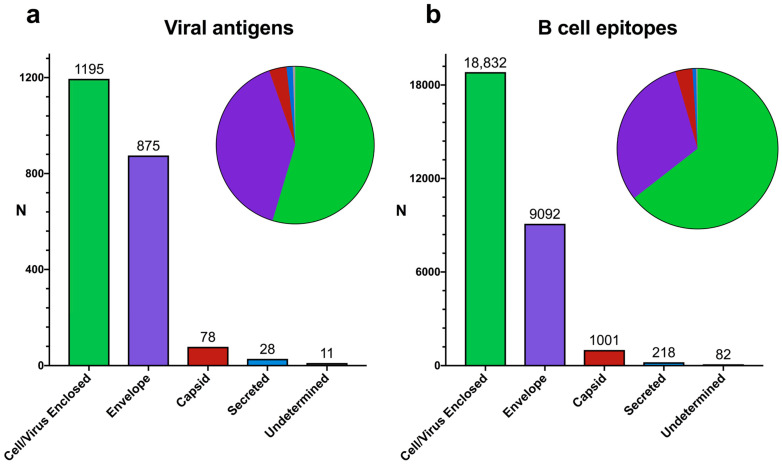
Classification of antigens and linear B cell epitopes from viruses. Bar plot depicting the number of viral antigens (**a**) and B cell epitopes (**b**) mapping to antigen groups, defined according to their visibility for antibody recognition: Cell/Virus Enclosed (in green, hidden); envelope (in violet, visible); capsid (in red, undetermined visibility); and secreted (in blue, visible). Viral antigens and their respective B cell epitopes that could not be assigned to a single group are classified as undetermined (in grey). Pie charts within bar plots illustrate the proportions of antigens and B cell epitopes in the different groups.

We were able to classify 2187 out of 3010 viral antigens into one of these groups according to the UNIPROT annotations, collectively encompassing 29,225 of the 48,377 selected linear B cell epitopes (Figure 2a). Hence, although considering antigens with UniProt annotations may lead to unexpected bias, the broader population of viral B cell epitopes was selected for further analysis. The B cell epitopes that were not classified into the aforementioned antigen groups corresponded to viral polyproteins and/or protein antigens without sub-cellular location annotations in the UniProt records. There were some overlaps in the classification of viral antigens but most of them were classified as Cell/Virus Enclosed (1195) and envelope (875) antigens (Figure 2a). These two groups of viral antigens encompassed 27,924 of the 29,225 B cell epitopes, distributed as follows: 18,832 B cell epitopes (64.44%) in Cell/Virus Enclosed antigens and 9092 epitopes (31.11%) in envelope antigens (Figure 2b).

**Figure 3:**
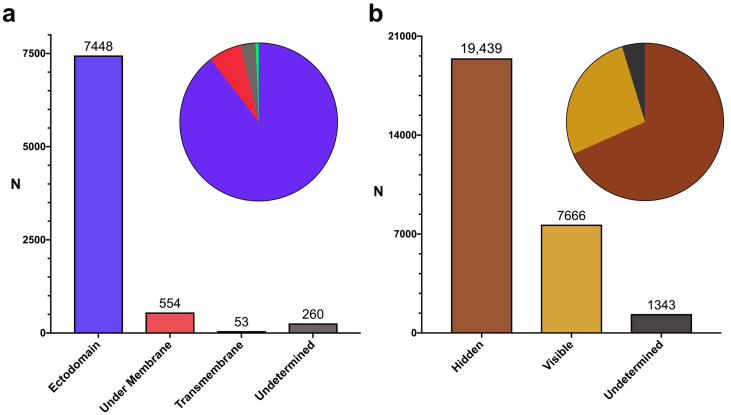
Analysis of B cell epitopes in viral antigens. (**a**) Bar plot depicting the number of viral envelope B cell epitopes mapping to distinct protein regions: ectodomain (purple), transmembrane (green), or under the plasmatic and/or envelope membrane (red). B cell epitopes that included segments mapping to more than one region are classified as undetermined (grey). (**b**) Bar plot depicting the number of virus-specific linear B cell epitopes classified according to their visibility. Hidden (brown): B cell epitopes in antigens enclosed in cells/viruses plus B cell epitopes in hidden regions of envelope proteins; visible (yellow): B cell epitopes in the ectodomains of envelope proteins and mature secreted proteins; undetermined (grey): B cell epitopes that could not be unequivocally classified as visible or hidden. In (**a**,**b**), pie charts within bar plots depict the proportions of B cell epitopes in the defined groups.

It is worth noting that, in envelope antigens, only the region surfacing the cell and/or the viral membrane (ectodomain) is readily visible for antibody recognition. Therefore, we obtained the topology of viral envelope antigens and subsequently identified the B cell epitopes located in ectodomains, transmembrane regions, and regions under the viral envelope membrane (under membrane), with the latter corresponding to cytosolic regions in the host cell. Of the 9092 linear B cell epitopes found in viral envelope proteins, 7448 resided in ectodomain regions and were readily visible to antibodies (Figure 3a). In this context, the total number of B cell epitopes in antigen regions that are hidden to antibody recognition rose to 19,439, corresponding to 68.33% of all virus-specific linear B cell epitopes that could be classified according to the visibility of their antigens (Figure 3b and Supplementary File S2).

### 3.3. Most Linear B Cell Epitopes Are Not Solvent Accessible in Native Antigens

**Figure 4:**
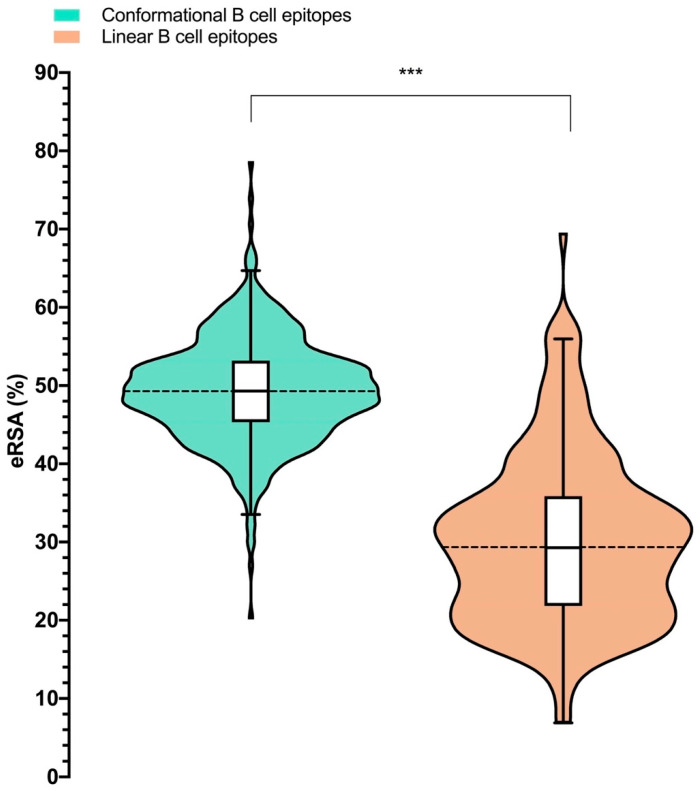
Solvent accessibility of B cell epitopes. Relative solvent accessibility of B cell epitopes (eRSA) was computed for linear (orange) and conformational (green) B cell epitopes and plotted as violin plots. Mann–Whitney U test was used to identify statistical differences between groups (*** *p*-value < 0.001).

B cell epitopes must also be solvent accessible to be recognized by antibodies. Therefore, we investigated whether potentially recognizable linear B cell epitopes (located in the ectodomains of viral envelope antigens and secreted antigens) were actually solvent accessible in the native 3D structure of the relevant antigens. To that end, we selected 220 linear B cell epitopes that mapped entirely to envelope antigens with available 3D structures. To each B cell epitope, we assigned an epitope relative solvent accessibility (eRSA) value, which was computed as the average of the relevant RSA values of the residues comprising the B cell epitope (Supplementary File S3). To establish a threshold of solvent accessibility, we carried out the same calculations for 496 conformational B cell epitopes (Supplementary File S4). As we show in Figure 4, the eRSA values of conformational B cell epitopes were significantly higher than those of linear B cell epitopes (*p*-value < 2.2·10^−16^). The median eRSA value of linear B cell epitopes was 29.34%, while the median eRSA value of conformational B cell epitopes was 49.29% (Figure 4). Using the minimum eRSA value (without outliers) of conformational B cell epitopes as a threshold (33.53%), we determined that 67.28% of linear B cell epitopes were not solvent accessible in the antigen 3D structure. It is worth noting that, in the calculation of solvent accessibility of linear B cell epitopes, we did not consider processes like the multimerization of antigens and protein dynamics, which can both affect solvent accessibility calculations. In particular, multimerization of protein antigens can decrease solvent accessibility, while protein dynamics could increase or decrease solvent accessibility. This RSA analysis may be biased by the limited availability of experimentally determined structures, and, arguably, expanding the analysis to predicted tertiary structures could have served to avoid such bias. Methods to predict tertiary structures have indeed become quite accurate. However, the prediction of tertiary structures has caveats and is time consuming. Moreover, the number of different protein folds is limited, and the experimental RSA analysis likely covers a large part of the antigens.

## 4. Discussion

**Figure 5:**
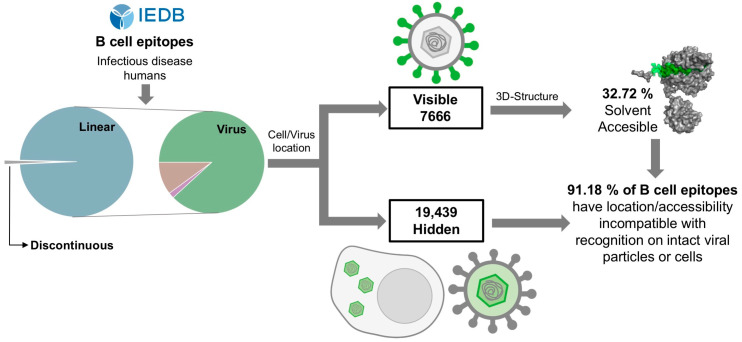
Schematic overview of the B cell epitope analysis. Virus-specific linear B cell epitopes were classified into antigen groups according to their visibility for antibody recognition. The solvent accessibility of B cell epitopes located in visible antigens was determined using available 3D structures. Overall, our findings indicate that most virus-specific linear B cell epitopes have a location and/or solvent accessibility that is incompatible with their recognition in native antigens on intact viral particles or cells.

B cell receptors and antibodies can only recognize visible and solvent-accessible antigens. Given the 3D structure of proteins, it is often assumed that most B cell epitopes in protein antigens are conformational, encompassing non-sequential residues that are in close proximity to the molecular surface of the antigen [4]. Currently, numerous B cell epitopes have been identified in different experimental settings and collected in dedicated databases, such as the IEDB, which is the largest repository. In this work, we sought to characterize the protein B cell epitopes available in the IEDB that are targeted in humans during infection, following the strategy depicted in Figure 5.

The overwhelming majority (86.94%) of the selected B cell epitopes were linear and from viruses (Figure 1). This result denotes that, on the one hand, researchers have had more success in determining the targets of antibody responses in viral infections than in infections caused by other pathogens; viruses are very simple organisms, and B cell epitope mapping studies are easier to conduct. An alternative interpretation for this result is that viral infections occur much more frequently than infections caused by other organisms; hence, antibody characterization studies are more likely to be performed on viruses. On the other hand, the result appears to contradict the assumption that most B cell epitopes are conformational. However, while this assumption applies to properly folded proteins, common approaches to B cell epitope mapping rely on detecting the binding of antibodies to antigen fragments (peptides) and hence yield only linear B cell epitopes. In any case, the detected linear B cell epitopes must have been visible and accessible for B cell recognition and elicitation of cognate antibodies during the course of infection, albeit not necessarily on intact antigens. Therefore, we investigated to what extent the reported virus-specific linear B cell epitopes are visible and accessible for antibody recognition on host cells and/or viral particles. To that end, we focused on a subset of linear B cell epitopes from viral antigens with UniProt annotations enabling such investigation.

We were surprised to find that most virus-specific B cell epitopes (64.44%) lay on viral antigens that remained enclosed in host cells or viral particles, precluding their recognition by antibodies in those contexts (Figure 2b). In comparison, linear B cell epitopes on envelope viral proteins represented only 31.11%. This percentage is surprisingly low considering the relevance of and interest in these antigens. Envelope proteins play key roles in viral entry into host cells and spreading infection, and are the main targets of neutralizing and anti-viral antibodies. Neutralizing antibodies, such as those against SARS-CoV-2 Spike protein, preclude binding to cognate host receptors, which is required for viral entry [19,20]. Non-neutralizing anti-viral antibodies prevent the function of envelope proteins required for spreading infection. For example, antibodies targeting the influenza A virus envelope protein neuraminidase limit infection because they can inhibit the enzymatic activity of neuraminidase, which is required for the virus to leave infected cells [21,22]. Interestingly, a substantial number of B cell epitopes in envelope proteins mapped outside of the ectodomain (6.68%), the protein region visible for antibody recognition (Figure 3a). Overall, we estimated that only 23.43% of virus-specific linear B cell epitopes targeted during the course of infection are in antigen regions visible for antibody recognition on viral particles and/or host cells (Figure 3b).

We also found evidence indicating that many virus-specific linear B cell epitopes in ectodomains of envelope antigens are not solvent-accessible. Analyzing linear B cell epitopes located in ectodomains with known 3D structures, we estimated that 67.28% of them have epitope relative solvent accessibilities (eRSA values) that are lower than the minimum eRSA determined for conformational B cell epitopes (Figure 4). Therefore, the majority of the examined B cell epitopes have solvent accessibilities that are incompatible with their recognition of native antigens, which likely holds true for all remaining linear B cell epitopes on envelope proteins. If this is the case, a mere 8.82% of all virus-specific B cell epitopes that are known to be targeted during infection could have been recognized on native antigens visible on viral particles and/or host cells (Figure 5).

That the vast majority of linear B cell epitopes have locations and solvent accessibilities unsuitable for antibody recognition in native antigens visible on viral particles and/or host cells implies that recognition must occur after the participation of processes that make them visible and accessible. One such process may be phagocytosis. Phagocyte cells such as neutrophils are recruited early on to the site of infection, where they engulf viral particles as well as dead infected cells that are killed by cytotoxic T cell lymphocytes (CTLs), or through antibody-dependent cellular cytotoxicity mediated by natural killer (NK) cells. CTLs and NK cells generally induce apoptosis in infected cells, but they can also contribute to the release of viral antigens that are hidden within the infected cells. Subsequently, phagocytes particulate and degrade the engulfed material in phagolysosomes, releasing their content via exocytosis to continue phagocyting [23,24,25,26]. Sentinel cells like dendritic cells and tissue-resident macrophages can also contribute to this process. Antigen degradation may further proceed in the extracellular space with the participation of different proteases, such as matrix metalloproteases [27,28] and proteases released by phagocytes [23,24,25,26]. As a result of all these processes, new antigens and antigen fragments are available for B cell recognition and the generation of specific antibodies. Clearly, once a protein becomes degraded, recognition of linear B cell epitopes becomes dominant.

It is legitimate to speculate about the functional purpose of generating so many antibodies against hidden antigens and epitopes that are unlikely to be protective. These non-protective antibodies may serve as “garbage-type” clean-up antibodies, helping phagocytes to clear cellular debris resulting from the destruction of infected cells. Moreover, antibodies and memory B cells elicited against antigen fragments are likely to be more cross-reactive than those elicited against conformational B cell epitopes and could be useful in heterologous viral infections by increasing inflammation—e.g., through complement activation and attraction of innate immune cells to the infection site—and delaying the spread of the virus until the onset of specific T cells and neutralizing antibodies. In this respect, we wonder if the recognition of so many linear B cell epitopes hidden from pathogens in early infections could be a strategy for the prompt elicitation of large numbers of antibodies and memory B cells that, through cross-reactivity, could be useful against unrelated infections. In fact, many potentially cross-reactive B cell epitopes can be identified between unrelated pathogens (e.g., between bacteria and viruses), which could support this possibility [29,30]. In any case, distinguishing protective specific antibodies from all the others should be considered a major task in immune diagnostics. Certainly, conventional assays using antigen fragments or antigens that could be, in part, denatured do not make this distinction.

## 5. Conclusions and Limitations

Our analysis of available B cell epitope data indicates that antibodies elicited during viral infections mostly target linear B cell epitopes requiring prior degradation of viral particles and/or infected cells. Hence, it demonstrates that B cell epitope and antibody repertoires are likely shaped by antigen degradation processes unconnected to presentation by major histocompatibility complex (MHC) molecules. Although we considered a vast set of virus-specific linear B cell epitopes, there are many distinct types of viruses, and whether our results and conclusions are generalizable to all of them, and to other pathogens, remains to be investigated. We are also aware that the scope of our conclusions could be limited by the fact that we assumed the locations of viral antigens based solely on UniProt sub-cellular locations, which may not reflect the dynamic nature of viral proteins. Likewise, the quality of the available B cell epitope data may also limit the scope of our findings. For example, we cannot rule out that discriminating B cell epitopes based on their binding affinity to antibodies somewhat affected our results. Unfortunately, the quantitative binding affinity of B cell epitopes to antibodies is not usually determined and/or reported. Likewise, because the information is not available, we do not know whether the B cell epitopes used in this study resulted from the engagement of naive B cells or pre-existing cross-reactive memory B cells.

## Data Availability

Data are contained within the article and supplementary materials.

## Supplementary Materials

The following supplementary materials are available online at https://www.mdpi.com/article/10.3390/cells13131076/s1. Supplementary File S1: linear B cell epitopes targeted during infection in humans; Supplementary File S2: virus-specific linear B cell epitopes, including their locations; Supplementary File S3: virus-specific linear B cell epitopes mapping to ectodomains of viral envelope proteins with available tertiary structures; Supplementary File S4: discontinuous B cell epitopes extracted from antigen–antibody tertiary structures.
